# Occurrence of Paraneoplastic Syndrome in Bronchogenic Carcinoma Patients in a Tertiary Care Centre in Chennai

**DOI:** 10.7759/cureus.82436

**Published:** 2025-04-17

**Authors:** Swathi Srinivas, Hariprasad Balakrishnan, Dhanasekar T

**Affiliations:** 1 Medicine, Sri Ramachandra Institute of Higher Education and Research, Chennai, IND; 2 Pulmonology, Sri Ramachandra Institute of Higher Education and Research, Chennai, IND

**Keywords:** bronchogenic carcinoma, non small cell lung cancer, paraneoplastic syndromes, small-cell lung carcinoma, tertiary care centre

## Abstract

Background

Bronchogenic carcinoma refers to tumors originating in the lung parenchyma or within the bronchi. Broadly, they are classified into small cell and non-small cell lung carcinoma. Non-small cell is further divided into adenocarcinoma, squamous cell carcinoma and large cell carcinoma. Paraneoplastic syndromes (PNS) are defined as the signs and symptoms attributed to cytokines or hormones released from a tumour or a patient's immune system. PNS is found to play a role in the prognosis of the patient, as well as in some cases, a preceding event before cancer diagnosis. Through this study, we aim to shed light on the occurrence of different paraneoplastic syndromes among the types of bronchogenic carcinoma.

Aim

To assess the occurrence of various paraneoplastic syndromes in patients diagnosed with bronchogenic carcinoma.

Materials and methods

In a prospective study at the Department of Respiratory Medicine at Sri Ramachandra Institute of Higher Education and Research, we explored the occurrence of paraneoplastic syndromes in patients with bronchogenic carcinoma. We calculated the sample size using n-Master software version 2.0 (https://nmaster.software.informer.com/).

Patients were classified based on the symptoms they experienced for neurological, musculoskeletal, hypercalcemia, Cushing syndrome, chronic gastrointestinal (GI) pseudo-obstruction, and limbic encephalitis. They were then graded based on the definitions of the new diagnostic criteria, 2021, as: Definite (high risk), Probable (intermediate risk), Possible (minimal risk), and No PNS. Further, the prevalence of PNS in each subtype of bronchogenic carcinoma was ascertained.

Results

The occurrence was found to be 35% in small cell; 28% in large cell; 20% in adenocarcinoma and 17% in squamous cell carcinoma. Further, Definite PNS was highest in small cell carcinoma, while No PNS was most prevalent in adenocarcinoma.

## Introduction

Small cell lung cancer accounts for about 15% of lung carcinomas and around 30,000 deaths in the United States annually. The median survival range is around 8-12 months [1]. A combination of complex pathophysiology, poor prognosis, and minimal therapeutic advances has led to the high mortality. Paraneoplastic syndromes are a group of clinical disorders that are associated with malignant diseases, but they are not directly related to the physical effects of the primary tumor [2]. These conditions can arise due to autoimmune reactions (immune cross reactivity between tumour and normal host tissue); humoral (secretion by tumours of functional peptides and hormones) or unknown [3].

The prevalence of paraneoplastic syndromes in bronchogenic carcinoma has been reported to be around 10% [4]. It depends on the histological type and stage of cancer, but it can also affect multiple organ systems at once. Certain subtypes are more commonly associated with paraneoplastic syndromes compared to others. Since their presence can significantly affect patient outcomes and prognosis, the early recognition of paraneoplastic syndromes in patients with bronchogenic carcinoma is crucial for prompt intervention and appropriate treatment.

Management involves treating the malignancy through surgery, chemotherapy with or without radiation therapy, along with supportive care aimed at the specific paraneoplastic syndrome. A comprehensive understanding of these syndromes and their association is essential for healthcare professionals to recognise them in time and provide effective management. This helps to plan an active intervention program for early treatment, improving quality of life and reducing morbidity. A comprehensive investigation into the prevalence of paraneoplastic syndromes in bronchogenic carcinoma has not been carried out in the Indian subcontinent, making this a pilot study.

## Materials and methods

A prospective study was conducted among patients diagnosed with bronchogenic carcinoma at the Department of Respiratory Medicine at Sri Ramachandra Institute of Higher Education and Research. We calculated the sample size using the following formula

\[ N = \frac{Z \cdot \alpha^2 \cdot p \cdot q}{L^2} \]

Where N is the number of patients, Z is the Z-score (2.576 for a 99% confidence interval), p is the estimated proportion of the population with the characteristic of interest, q=1-p, and L is the margin of error (0.05).

Ethical approval for the study was obtained from the Institutional Ethics Committee at Sri Ramachandra Institute of Higher Education and Research (IEC approval number- CSP/23/APR/126/282), and informed consent was also obtained. The study population was a group of 166 patients diagnosed with bronchogenic carcinoma from June 2023 to May 2024, attending a tertiary care centre. They were examined further to note for signs of PNS. The minimum number of samples to conduct this study was 60.

A case proforma was prepared to collect data. Investigations done for every recruited patient include X-ray chest, CT chest, biopsy, and histopathology of the specimen, as well as immunohistochemistry. The patients were subjected to the proforma and were classified based on the symptoms they experienced for neurological, musculoskeletal, hypercalcemia, Cushing syndrome, chronic GI pseudo-obstruction, and limbic encephalitis. They were then graded based on the definitions of the new diagnostic criteria, 2021, as: Definite (high risk), Probable (intermediate risk), Possible (minimal risk), and No PNS. The category depended on multiple factors like age, gender, Charlson’s comorbidity index, and the various symptoms experienced.

Charlson’s comorbidity index was graded as no comorbidity, low comorbidity, and high comorbidity based on the presence of conditions like COPD, non-insulin dependent diabetes mellitus (DM), hypertension, Ischemic heart disease, and kidney disease. This is an important prognostic factor. Finally, the risk of PNS was ascertained based on the new diagnostic criteria that classified 26-30 points as high risk (Definite), 12-24 points as intermediate risk (Probable), 1-12 points as minimal risk (Possible), and 0 points as no risk. Further, the occurrence of PNS among different types of bronchogenic carcinoma was also ascertained. Data collection was done by the principal investigator, and SPSS 16.0 software (SPSS Inc., Chicago) was used for data analysis.

## Results

Over the duration of one year, we identified 166 patients who satisfied the inclusion criteria. Out of the total, 100 (60.2%) patients with small cell carcinoma, 15 (9%) patients with large cell carcinoma, 35 (21.08%) patients with squamous cell carcinoma, and 16 (9.63%) patients with adenocarcinoma were recruited for the study. The duration since diagnosis for all patients was less than one year. Only patients who were diagnosed from January 2023 to December 2023 were considered.

Table 1 shows that small cell carcinoma was the most common subtype of bronchogenic carcinoma, followed by squamous cell carcinoma, and large cell carcinoma was the least common. Out of these patients, two (1.20%) had a Charlson’s comorbidity index of 1, 99 (59.63%) patients had an index of 1-2, and 65 (39.15%) patients had an index of 3+.

**Table 1:** Population cohort of patients diagnosed with bronchogenic carcinoma represented as N (and % in parenthesis)

Characteristics	Small cell carcinoma	Large cell carcinoma	Squamous carcinoma	Adenocarcinoma
	N	N	N	N
	Total	100 (60.2%)	15 (9.03%)	35 (21.08%)	16 (9.63%)
Age (years) at index date				
20-30	1 (0.60%)	0 (0%)	0 (0%)	1 (0.60%)
30-40	4 (2.40%)	3 (1.80%)	3 (1.80%)	0 (0%)
41-50	10 (6.02%)	2 (1.20%)	8 (4.81%)	1 (0.60%)
51-60	33 (19.80%)	5 (3.01%)	9 (5.42%)	8 (4.81%)
61-70	34 (20.48%)	3 (1.80%)	10 (6.02%)	5 (3.01%)
71-80	18 (10.84%)	2 (1.20%)	5 (3.01%)	1 (0.60%)
Sex				
Male	65 (39.15%)	11 (6.62%)	21 (12.65%)	10 (6.02%)
Female	35 (21.08%)	4 (2.40%)	14 (8.43%)	6 (3.61%)
Charlson’s comorbidity index,				
0	1 (0.60%)	0 (0%)	0 (0%)	1 (0.60%)
1-2	58 (34.93%)	8 (4.81%)	21 (12.65%)	12 (7.22%)
3+	41 (24.69%)	7 (4.21%)	14 (8.43%)	3 (1.80%)

The occurrence of paraneoplastic syndromes was found to be 35% (N=58) in small cell carcinoma, 28% (N=45) in large cell carcinoma, 20% (N=34) in adenocarcinoma, and 17% (N=29) in squamous cell carcinoma. Table 1 shows the population cohort of patients included in the study.

Among the different categories of paraneoplastic syndromes, neurological and limbic encephalitis were the two most commonly occurring types. Occurrence of neurological PNS was 93% (N=93) in small cell carcinoma, 53.3% (N=8) in large cell carcinoma, 31.4% (N=11) in squamous cell carcinoma and 18.75% (N=3) adenocarcinoma, while the prevalence of limbic encephalitis was 97% (N=97) in small cell carcinoma, 40%(N=6) in large cell carcinoma, 25.7% (N=9) in squamous cell carcinoma and 31.25%(N=5) in adenocarcinoma. Table 2 shows the prevalence of different types of paraneoplastic syndromes among subtypes of primary bronchogenic carcinoma.

**Table 2:** Occurrence of paraneoplastic syndromes among types of primary bronchogenic carcinoma represented as N (and % in parenthesis) Significant p-value according to Chi-square test is p<0.05. PNS: Paraneoplastic syndromes, GIT: Gastrointestinal tract.

Condition	Small cell carcinoma N= 100	Large cell carcinoma N= 15	Squamous carcinoma N= 35	Adenocarcinoma N= 16	Chi square value	p-value
All potential PNS	99 (59.63%)	13 (7.83%)	18 (10.84%)	6 (3.61%)	63.23	1.20e-13
Neurological	93 (56.02%)	8 (4.81%)	11 (6.62%)	3 (1.80%)	70.28	2.63e-15
Musculoskeletal	82 (49.39%)	8 (4.81%)	1 (0.60%)	5 (3.01%)	72.09	1.52e-15
Hypercalcemia	61 (36.74%)	8 (4.81%)	2 (1.20%)	2 (1.20%)	39.53	1.34e-08
Cushing syndrome	62 (37.34%)	6 (3.61%)	5 (3.01%)	0 (0%)	38.36	2.37e-08
Chronic GIT pseudo obstruction	5 (3.01%)	8 (4.81%)	1 (0.60%)	3 (1.80%)	36.63	5.52e-08
Limbic encephalitis	97 (58.43%)	6 (3.61%)	9 (5.42%)	5 (3.01%)	86.05	1.54e-18

According to the latest diagnostic criteria in 2021, Definite and Intermediate PNS were most commonly identified in small cell carcinoma, Minimal PNS was most common in large cell carcinoma and No PNS was most common in adenocarcinoma. From Table 1, small cell carcinoma was the most common subtype of bronchogenic carcinoma, followed by squamous cell carcinoma and large cell carcinoma being the least common.

**Figure 1:**
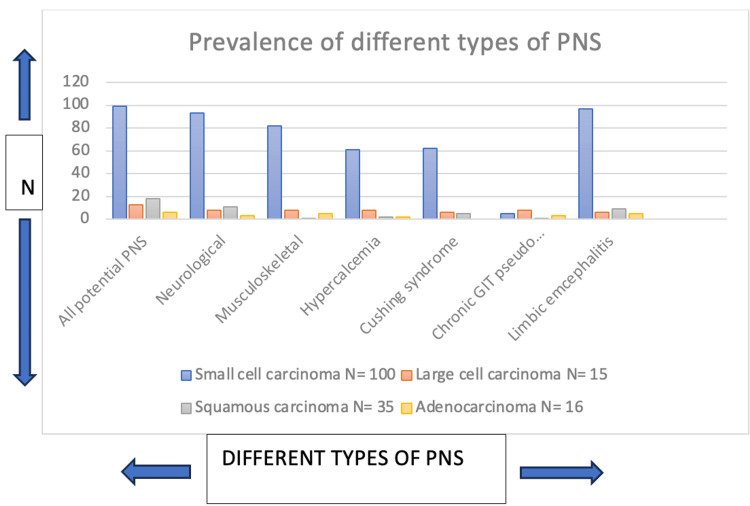
Occurrence of different types of paraneoplastic syndromes

From Figure 1, it is concluded that among the paraneoplastic syndromes, neurological and limbic encephalitis emerged as the two most common categories. Paraneoplastic syndromes (PNS) were most prevalent in small cell carcinoma, followed by large cell carcinoma and adenocarcinoma, squamous cell carcinoma exhibited the lowest prevalence of PNS.

**Figure 2:**
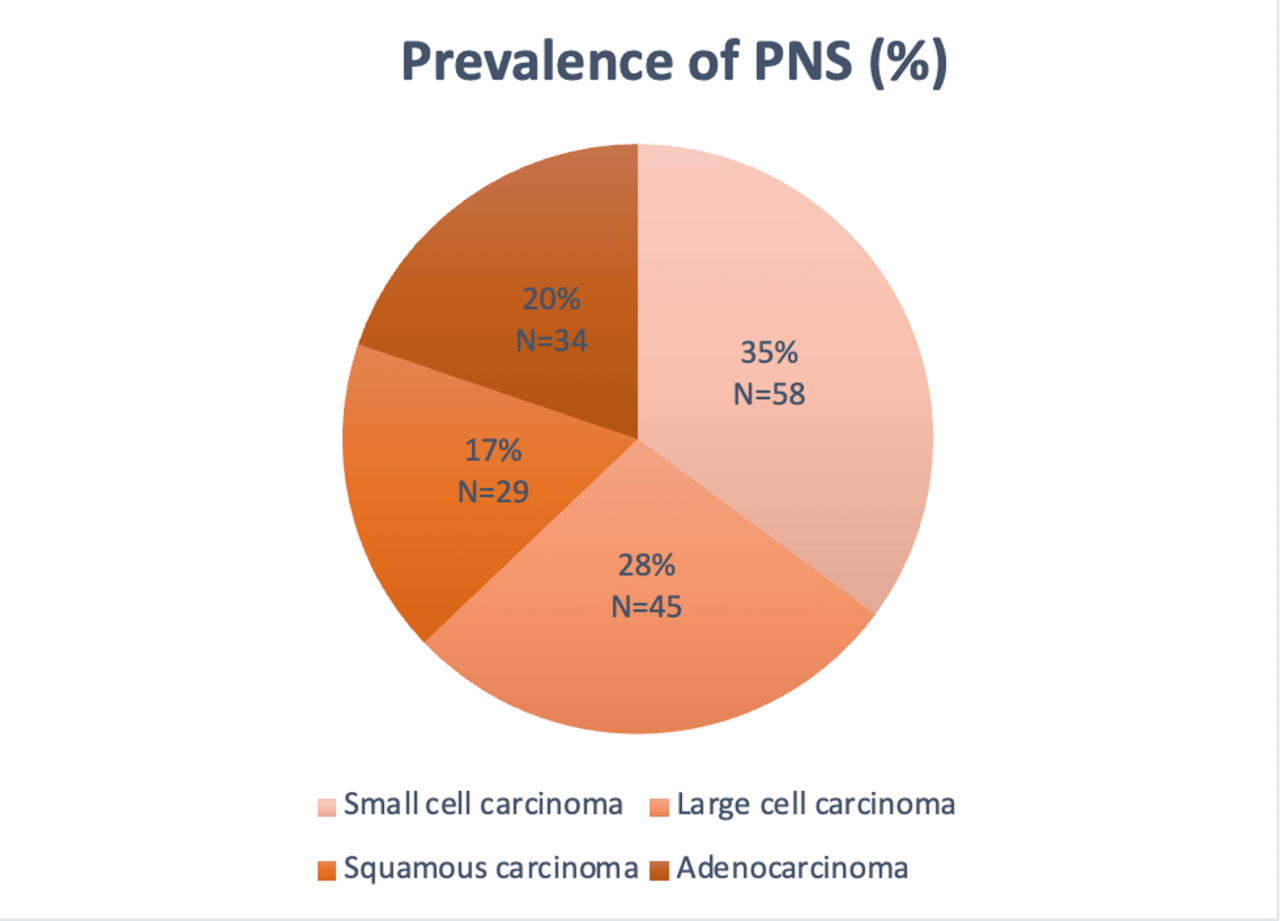
Occurrence of paraneoplastic syndromes among different subtypes of bronchogenic carcinoma

Figure 2 shows that paraneoplastic syndromes (PNS) were most prevalent in small cell carcinoma, followed by large cell carcinoma and adenocarcinoma; squamous cell carcinoma exhibited the lowest prevalence of PNS. The study reported specific prevalence rates, revealing that PNS occurred in 35% (N=58) of small cell carcinoma cases, 28% (N=45) in large cell carcinoma, 20%(N=34) in adenocarcinoma, and 17%(N=29) in squamous cell carcinoma.

**Figure 3:**
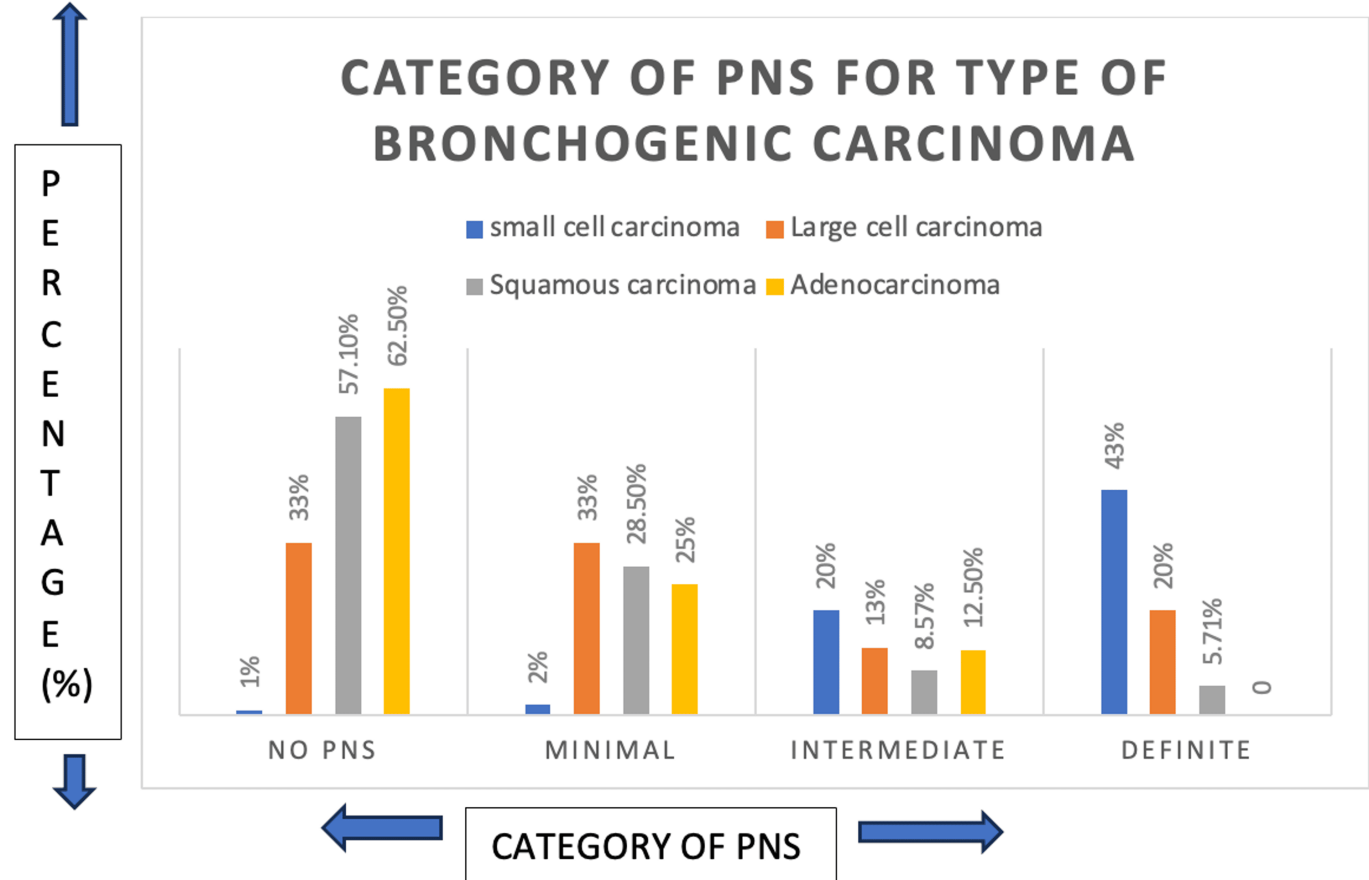
Categories of paraneoplastic syndromes for different types of bronchogenic carcinoma These categories are according to the latest diagnostic criteria, 2021.

The analysis in Figure 3 indicates that definite PNS was most frequently associated with small cell carcinoma, while no PNS was most prevalent in adenocarcinoma.

## Discussion

To the best of our knowledge, this is the first prospective study to explore the occurrence of paraneoplastic syndromes in bronchogenic carcinoma by applying the latest diagnostic criteria to the Indian population. Over the past few decades, there has been a rise in the incidence of bronchogenic carcinoma. It is the most common cancer in men worldwide, with an age-standardized rate (ASR) of 33.8 per 100,000, and it is the fourth most frequent cancer in women (13.5 per 100,000) [5]. Cigarette smoking is the most common risk factor for bronchogenic carcinoma. It is estimated that approximately 90% of lung cancer deaths in men and 80% of lung cancer deaths in women around the world each year are caused by smoking [6].

Other risk factors include secondhand smoking, radon exposure, occupational exposure to carcinogens like asbestos, and other environmental agents [7]. In this setting, it is imperative to identify and treat bronchogenic carcinoma early on to improve survival rates and the quality of life. Current trends show that the prevalence of paraneoplastic syndromes in bronchogenic carcinoma is significant. They may manifest either prior to or following the diagnosis of bronchogenic carcinoma. Thus, the identification and management of PNS is important to improve patient outcomes.

The pathophysiology involves the production of bioactive substances by the tumor cells, which can affect distant tissues and organs. These substances are often hormones and cytokines, which lead to various systemic manifestations that are independent of the local effects of the primary tumour. The immune system may also play a role in the development of PNS, as the body’s response to the tumor can lead to autoimmune reactions and production of antibodies that cross-react with normal tissues. The exact mechanisms can vary depending on the specific paraneoplastic syndrome associated with bronchogenic carcinoma [8].

They can manifest as neurological, musculoskeletal, hypercalcemia, Cushing syndrome, chronic gastrointestinal pseudo-obstruction, or limbic encephalitis. Neurological challenges such as difficulty walking or maintaining balance, diminished muscle coordination or tone, impaired fine motor skills, and difficulty in swallowing or slurring of speech. Musculoskeletal PNS takes into account fatigue, arthralgia, myalgia, and acanthosis nigricans. Hypercalcemia is characterized by the presence of thirst, polyuria, dehydration, anorexia, vomiting, or abdominal pain. Cushing syndrome takes into account Moon facies, acne, purple striae, proximal muscle weakness, peripheral oedema, or hypertension. Chronic gastrointestinal pseudo-obstruction considers abdominal pain, nausea, vomiting, or severe constipation, and limbic encephalitis takes into account personality changes, irritability, depression, seizures, memory loss, confusion, or dementia [3].

Our studies show that PNS can be more prevalent in small cell carcinoma than other types of bronchogenic carcinoma, which is consistent with other similar studies. Miret et al. suggest that PNS was eight-fold more common in small cell carcinoma and four-fold more common in non-small cell carcinoma compared to the general population [9].

In our studies, 166 patients of different subtypes of bronchogenic carcinoma were studied to identify the occurrence of paraneoplastic syndromes in various histological types. Of these, 60% (N=100) of patients belonged to the small cell carcinoma subtype, followed by squamous cell carcinoma (21%, N=35), adenocarcinoma (9.6%, N=16), and large cell carcinoma (9%, N=15). These findings are in agreement with the known epidemiological distribution of bronchogenic carcinoma, in which small cell carcinoma often presents more aggressively and has a higher association rate with PNS. The occurrence of PNS varied considerably amongst carcinoma subtypes. It was highest for small cell carcinoma at 35% (N=58), an already known characteristic of this aggressive tumour type, associated with high neuroendocrine activity. In contrast, large cell carcinoma had a PNS prevalence of 28% (N=45), adenocarcinoma 20% (N=34), and squamous cell carcinoma the lowest at 17% (N=29). These findings coincide with the current understanding that the PNS, especially neurological syndromes, are associated more often with tumours of neuroendocrine origin, such as small cell carcinoma. Neurological PNS were found in 93% (N=93) of small cell carcinoma cases, far outweighing the rest of the subtypes. Large cell carcinoma follows in neurological PNS prevalence at 53.3% (N=8), while that of squamous cell carcinoma and adenocarcinoma was considerably lower at 31.4% (N=11) and 18.75% (N=3), respectively. This result is important, as it confirms the close association between small cell carcinoma and neurological PNS as a clinical fingerprint for this carcinoma type. The proportion of limbic encephalitis as a serious form of neurological PNS also reached a peak in small cell carcinoma at 97% (N=97). The neuroendocrine features of small cell carcinoma typically provoke an autoimmune reaction that impacts the nervous system. The prevalence was 40% (N=6) in large cell carcinoma, while that for squamous cell carcinoma and adenocarcinoma was lower: 25.7% (N=9) and 31.25% (N=5), respectively. This high incidence in small cell carcinoma patients suggests that early screening for neurological symptoms in this group is called for, since in general, the better the timing of identification and treatment of PNS, the better the outcomes. According to Charlson's comorbidity index, the majority had moderate to severe comorbidities; 99 (59.63%) had a score within a range of 1-2, while 65 (39.15%) scored 3 and above. Only two (1.20%) had a score of 1, showing that this cohort generally had a high burden of comorbidities. While the direct relationship between comorbidities and the onset of PNS was not studied, it would be of interest for future research to establish if higher comorbidity indices are indeed associated with a worse prognosis in cancer patients with PNS.

Diagnosis of PNS can be challenging due to the nonspecific symptoms in the setting of bronchogenic carcinoma. The process involves a combination of clinical assessments through history and examination, along with laboratory tests that identify autoantibodies or serum markers. Other modalities include electromyography and biopsy of tissue [10]. The treatment involves symptomatic management depending on the type of manifestation. Primary treatment is to treat the underlying bronchogenic carcinoma through surgical resection, chemotherapy, and radiotherapy. Second-line management involves immunotherapy, corticosteroids, plasmapheresis, and regular monitoring [10].

The impact of PNS on the overall prognosis of bronchogenic carcinoma is significant and can be influenced by various factors such as type and severity of PNS, underlying cancer stage, presence of metastasis, effectiveness of treatment, histological type of bronchogenic carcinoma, age of the patient, and immunological factors. At later stages, PNS can be very extensive and have severe manifestations, whereas in earlier stages, the expression of symptoms will be mild [11].

Identification of PNS also holds prognostic value. In some cases, since PNS precedes cancer diagnosis, identification of PNS can also lead to an early diagnosis of carcinoma and thus a better prognosis. This has even been shown to increase the survival rate of the patient [11]. Thus, the early recognition of paraneoplastic syndromes is important in the clinical management of bronchogenic carcinoma patients, in terms of diagnosis, prognosis, and treatment. It is important to create awareness among medical professionals on the significance of paraneoplastic syndromes in the overall management to improve the quality of life for the patient.

Limitations

(1) The study population was limited to a single tertiary care centre. (2) There was a reporting bias from the end-stage patients. (3) There was a heterogeneity of bronchogenic carcinoma and a lack of an equal number of participants from each type.

## Conclusions

In conclusion, our study sheds light on the occurrence of paraneoplastic syndromes in bronchogenic carcinoma, providing an insight into the relationship between these syndromes and bronchogenic carcinoma. Our analysis revealed a noteworthy occurrence of paraneoplastic syndromes in patients with bronchogenic carcinoma, highlighting the importance of considering these manifestations in the clinical evaluation and management of patients. Early detection and recognition of paraneoplastic syndromes can enhance diagnostic precision, guide more targeted therapeutic interventions, and even develop a personalized approach for the diagnosis and management of bronchogenic carcinoma and its associated paraneoplastic syndromes.

Research focused on the occurrence of paraneoplastic syndromes in bronchogenic carcinoma contributes to advancements in early detection, targeted treatment, and improved patient outcomes. In the future, investigating the molecular mechanisms for the development of PNS can provide intricate insights into the pathophysiology of PNS and, therefore, prompt treatment. Clinical trials can be conducted to develop novel therapeutic approaches for bronchogenic carcinoma associated with paraneoplastic syndromes. This is vital for devising new diagnostic methods and advancing personalized medicine.

## Appendices

Participant forms:

**Table 3:** Initial information

Parameter	Answer
Name of the patient	
Gender	
Age	
Date of diagnosis	
Diagnostic method	

**Table 4:** Assess comorbidities and calculate Charlson's comorbidity Index

Parameter	Answer
COPD	Yes/No
Non insulin dependent DM	Yes/No
Hypertension	Yes/No
Ischemic heart disease	Yes/No
Kidney disease	Yes/No
Charloson comorbidity index	0, 1-2, 3+

**Table 5:** Assess symptoms for different paraneoplastic syndromes that are commonly known to occur with bronchogenic carcinoma

Parameter	Answer
Neurological	Difficulty in walking Difficulty in maintaining balance Loss of muscle coordination Loss of muscle tone Loss of fine motor skills Difficulty in swallowing Slurred speech None
Musculoskeletal	Fatigue Arthralgia Myalgia Acanthosis nigricans None
Hypercalcemia	Thirst Polyuria Dehydration Anorexia Vomiting Abdominal pain None
Cushing syndrome	Moon facies Acne Purple striae Proximal muscle weakness Peripheral edema Hypertension None
Chronic GI pseudo obstruction	Abdominal pain Nausea Vomiting Severe constipation None
Limbic encephalitis	Personality changes Irritability Depression Seizures Memory loss Confusion Dementia None

**Table 6:** Collects additional investigation findings and calculates the final paraneoplastic syndromes score

Parameter	Answer
Significant chest xray findings	
Significant CT findings	
Significant HPE findings	
Significant IHC findings	
Type of lung malignancy	Small cell; large cell; squamous; adenocarcinoma
Total PNS score	
Risk	High risk (definite): 24-30 Intermediate risk (Probable): 13-24 Minimal risk (Possible): 1-12 No PNS: 0
